# Comparison of Stress-Induced Changes in Adults and Pups: Is Aldosterone the Main Adrenocortical Stress Hormone during the Perinatal Period in Rats?

**DOI:** 10.1371/journal.pone.0072313

**Published:** 2013-09-05

**Authors:** János Varga, Szilamér Ferenczi, Krisztina J. Kovács, Alexandra Garafova, Daniela Jezova, Dóra Zelena

**Affiliations:** 1 Department of Behavioral Neurobiology, Institute of Experimental Medicine, Budapest, Hungary; 2 Laboratory of Molecular Neuroendocrinology, Institute of Experimental Medicine, Budapest, Hungary; 3 Laboratory of Pharmacological Neuroendocrinology, Institute of Experimental Endocrinology, Slovak Academy of Sciences, Bratislava, Slovakia; John Hopkins University School of Medicine, United States of America

## Abstract

Positive developmental impact of low stress-induced glucocorticoid levels in early development has been recognized for a long time, while possible involvement of mineralocorticoids in the stress response during the perinatal period has been neglected. The present study aimed at verifying the hypothesis that balance between stress-induced glucocorticoid and mineralocorticoid levels is changing during postnatal development. Hormone responses to two different stressors (insulin-induced hypoglycaemia and immune challenge induced by bacterial lipopolysaccharid) measured in 10-day-old rats were compared to those in adults. In pups corticosterone responses to both stressors were significantly lower than in adults, which corresponded well with the stress hyporesponsive period. Importantly, stress-induced elevations in aldosterone concentration were significantly higher in pups compared both to corticosterone elevations and to those in adulthood with comparable adrenocorticotropin concentrations in the two age groups. Greater importance of mineralocorticoids compared to glucocorticoids in postnatal period is further supported by changes in gene expression and protein levels of gluco- (GR) and mineralocorticoid receptors (MR) and selected enzymes measured by quantitative PCR and immunohystochemistry in the hypothalamus, hippocampus, prefrontal cortex, liver and kidney. Gene expression of 11beta-hydroxysteroid dehydrogenase 2 (11β-HSD2), an enzyme enabling preferential effects of aldosterone on mineralocorticoid receptors, was higher in 10-day-old pups compared to adult animals. On the contrary, the expression and protein levels of GR, MR and 11β-HSD1 were decreased. Presented results clearly show higher stress-induced release of aldosterone in pups compared to adults and strongly suggest greater importance of mineralocorticoids compared to glucocorticoids in stress during the postnatal period.

## Introduction

Aldosterone is the main mineralocorticoid hormone involved in the control of water-electrolyte balance [1]. Together with glucocorticoids, aldosterone is released from the adrenal cortex in response to several stress stimuli [2]. Stimulation of aldosterone release by acute and chronic stressors is mediated by angiotensin II and adrenocorticotropic hormone (ACTH) [3], [4].

In stress research, less attention has been given to aldosterone compared to cortisol and corticosterone (the main glucocorticoids in humans and rodents, respectively) mainly because they are acting on the same receptors and glucocorticoids are the dominant players. They have two types of receptors, namely the low affinity glucocorticoid receptor (GR) and the high affinity mineralocorticoid receptor (MR) [5]. Thus, GRs are activated when glucocorticoid concentrations are high as occurs during stress or at the peak of the circadian rhythm, while MRs are thought to be close to saturation at baseline, non-stress conditions and exhibit tonic influence on hypothalamo-pituitary-adrenocortical axis (HPA) output [6], [7].

Since concentration of circulating glucocorticoids is 2–3 order higher than that of aldosterone, in aldosterone target tissues such as in the kidney, only the presence of 11-beta-hydroxysteroid dehydrogenase type 2 (11β-HSD2) allows steroid binding to receptors via conversion the competing corticosterone and cortisol into inactive metabolites [8]. On the other hand, 11β-HSD1 catalyses an opposite reaction by increasing active glucocorticoids in their target tissues (e.g. liver, brain).

Effects of stress-induced aldosterone release on the salt-water homeostasis have impact on the control of blood pressure and cardiovascular functions as well [9]. Recent findings revealed that the physiological and pathophysiological role of aldosterone may be much broader. Although brain MRs are related to behavioral expressions of mood, the action of the mineralocorticoid hormones in this respect was neglected due to very low levels of the enzyme 11β-HSD2 [7]. However, evidence is accumulating that certain brain regions contain MRs that bind preferentially mineralocorticoids [10]. Indeed, anxiogenic and depressogenic effects of aldosterone have been recently described [11]–[13].

Another important issue could be the involvement of aldosterone in neuroendocrine responses to stress stimuli during the postnatal period. Even though there are reports indicating that postnatal stress may have implications on the salt intake in the adulthood [14], this topic has been mostly neglected. During the first two weeks of life (from about days 4 to 14) rat pups show reduced capacity to secrete corticosterone in response to several stimuli [15]–[17]. This period has been termed the stress hyporesponsive period (SHRP). Because of the serious long-term consequences of high glucocorticoid levels during the perinatal period (e.g. hypertension, hyperlipidaemia, diabetes [18], [19]) it is crucial to maintain their levels at minimum. Though the low total corticosterone concentrations in pups are partially offset by low plasma corticosteroid-binding globulin (CBG) levels and its reduction during stress [20], [21], the time and stressor specific glucocorticoid hyporesponsiveness is still present not only in rodents [15], [22], but also in humans [23] and other vertebrates [24]. At the same time, hormone responses and coping with aversive stress stimuli are indispensable to life.

In the present studies, we aimed to test the hypothesis that the balance between stress-induced glucocorticoid and mineralocorticoid levels is changing during development. More specifically, we hypothesized that during SHRP, which is associated with decreased glucocorticoid secretion, mineralocorticoids are the dominant adrenocortical steroids released during stress.

## Materials and Methods

### Animals

Adult (265–415 g, 10–12-week-old) and postnatal (28–31 g, 10-day-old) male Wistar rats were investigated (Charles River, Hungary). Rats were kept in controlled environment (23±1°C, 50–70% humidity, 12 h light starting at 07∶00 h) and given commercial rat chow (Charles River, Budapest, Hungary) and tap water ad libitum.

### Ethics Statement

The experiments were performed in accordance with the European Communities Council Directive of November 24, 1986 (86/609/EEC), and were reviewed and approved by the Animal Welfare Committee of the Institute of Experimental Medicine (MÁB 22.1/2654/003/2007).

### Stress models

#### Insulin-induced hypoglycaemia

Stress of hypoglycaemia was induced in fasted rats by intraperitoneal (i.p.) insulin injection (Actrapid, Novo Nordisk, Bagsvaerd, Denmark) at the dose of 3IU/1 ml/kg of body weight in saline. Adult rats were fasted for 18 h, while 10-day-old pups for only 4 h (the entire litter was removed from the dam and put into a new cage with bedding and external heating) to achieve comparable degree of starvation. Rats of control groups were injected with the same volume of vehicle (0.9% NaCl). Separate groups of rats were decapitated at 60 and 90 min after i.p. injection. As the HPA axis of pups reacts to different stimuli with a delay, we took the 90 min results as more representative and presented these results in the graphic form. Data obtained at 60 min are in a table. Blood glucose levels were measured by commercially available analyser (D-Cont Personal, 77 Elektronika Kft., Budapest, Hungary) at the time of decapitation.

#### Immune challenge induced by lipopolysaccharide (LPS) injection

Administration of LPS represents a strong stressor simulating bacterial infection. Escherichia coli LPS, serotype 0111: B4 (Sigma, Budapest, Hungary) was used at the dose of 100 μg/1 ml/kg of body weight i.p. Rats of control groups were injected with the same volume of vehicle (0.9% NaCl). Animals were killed by decapitation 120 min after the injection.

### Sensitivity of adrenal cortex to ACTH in vitro

Incubation followed the method of Stachura et al. [25]. Each adrenal gland was chopped into small pieces with a sterile scalpel blade and preincubated in 1 ml DMEM at 37°C under 95% O_2_–5% CO_2_ atmosphere for 60 min. Thereafter the buffer was replaced with fresh DMEM, and the adrenals were preincubated for additional 60 min. Following the preincubation, 15-min samples were collected three times adding 10^−10^ M ACTH into the second fraction. At the conclusion of the experiment, media were removed, centrifuged at 3000 g for 5 min and the supernatant was stored at −20°C until corticosterone and aldosterone measurements. The total secreted amount during the whole observation period (3×15 min) was expressed as area under the curve (AUC).

### Hormone assays

Trunk blood was collected by decapitation into ice-cold plastic tubes, centrifugated at 2000 g for 20 min at 4°C and the serum was stored at −20°C until analysed. Because of the small amount of blood in postnatal rats, separate pups were used for ACTH/corticosterone and for plasma renin activity/aldosterone measurements. Samples from a particular experiment were always assessed in the same RIA.

Plasma ACTH concentrations were measured by radioimmunoassay (RIA) in 50 μl of unextracted plasma as described earlier [26]. The intraassay coefficient of variation was 4.7%. Concentrations of plasma corticosterone were measured in 10 μl of unextracted plasma by RIA as described earlier [27]. The intraassay coefficient of variation was 12.3%. Plasma aldosterone levels and plasma renin activity were measured using RIA Aldosterone kit and Angiotensin I RIA kit (Immunotech, France) [28]. The intrassay coefficient of variation was 9.5%. The same RIAs were used to measure corticosterone and aldosterone concentrations in the incubation medias.

### Measurements of gene expression of selected receptors and enzymes

Tissue samples of macrodissected brain regions, kidney and liver were collected under RNase free conditions from unstressed adult and postnatal rats and were kept at −70°C. Total RNA was isolated from homogenates of the hypothalamus, pituitary, hippocampus, prefrontal cortex, kidney and liver using RNeasy Mini Kit (Qiagen, Valencia, CA, USA) and then converted to cDNA by High-Capacity cDNA Reverse Transcription Kit (Life Technologies, Foster City, CA, USA). The cDNA samples were pooled for each group and measured in duplicates. Real-time PCR was performed using Power SYBR Green PCR Master Mix (Life Technologies) on ABI StepOnePlus instrument according to the manufacturer's instructions. Primers used for the comparative *C*_T_ experiments were designed by the Primer Express 3.0 program. Melting curve analysis to confirm the identity of PCR products has been performed by using ABI StepOnePlus instrument's Software v.2.1 according to the instructions of the manufacturer. Gene expression was analyzed by ABI StepOne Software 2.1 program. The primers were the following:

GR forward: 5′-CAT CTT CAG AAC AGC AAA ATC GA-3′, reverse: 5′-AGG TGC TTT GGT CTG TGG GAT A-3′; MR forward: 5′-CCA AGG TAC TTC CAG GAT TTA AAA AC-3′, reverse: 5′-AAC GAT GAT AGA CAC ATC CAA GAA TAC T-3′; 11β-HSD1 forward: 5′-CCT CCA TGG CTG GGA AAA T-3′, reverse: 5′-AAA GAA CCC ATC CAG AGC AAA C-3′; 11β-HSD2 forward: 5′-CGC CGC TTC CTA CAG AAC TT-3′, reverse: 5′-TCC TGG GTT GTG TCA TGA ACA-3′; GAPDH forward: 5′-ACA GCC GCA TCT TCT TGT GC-3′, reverse: 5′-GCC TCA CCC CAT TTG ATG TT-3′.

GAPDH was used as endogenous control. Relative quantity of mRNAs was referred to corresponding samples of the adult Wistar rats based upon the 2^−ΔΔCT^ method.

### Immunohistochemistry

Animals were anesthetized at rest by pentobarbital (50 mg/kg intraperitoneal) and perfused transcardially with saline solution (0.9% NaCl) for 2 min, then with 30 mL (pups) or 300 mL (adult) ice cold fixative (4% paraformaldehyde in 0.1 M Borate buffer pH 8). The brains were removed, post-fixed in the fixative for 3 h then placed in PBS 10% sucrose overnight at 4°C. On the next day 30 μm sections were cut in the coronal plane on a freezing microtome and were stored at −20°C in cryoprotectant. The sections were washed first in PBS for 3×10 min and for blocking the endogen peroxidase in H_2_O_2_ solution and again 3×5 min in PBS. Then sections were incubated in 2% Normal Goat Serum (Vector, Burlingame, USA) for 60 min. The GR and MR proteins were immuno labeled with rabbit polyclonal antibodies raised against GR and MR (Santa Cruz Biotechnology, USA) for 48 h at 4°C. This was followed by incubation in biotinylated anti-rabbit serum (1∶500; Vector, Burlingame, USA). The antigens were then visualized by conventional Avidin-Biotin-HRP technology (ABC, Vestastain; 1∶1000 TRIS) and developed using 0.05% diaminobenzidine (DAB, Sigma) and 0.01% H_2_O_2_ in PBS. Sections were then mounted onto geletine coated slides, dehydrated and coverslipped. Images were taken with a digital camera (NIKON, DMX 1200) coupled to a bright-field microscope (NIKON, Eclipse E400), using a 20× objective, with no further modifications. The following brain areas were investigated: hypothalamus (paraventricular nucleus and medial basal hypothalamus), prefrontal cortex, hippocampus. Representative photomicrographs are shown on figures.

### Statistical analysis

Values are presented as mean ± SEM. Data were analysed by analysis of variance using one (age) or two way ANOVA (age, treatment) of the STATISTICA 11.0 software package (StatSoft Inc. Tulsa, Okla, US). Multiple pairwise comparisons where appropriate were made by the Newman–Keuls method. Results of the post hoc analysis are presented on the figures. In case there was no significant interaction, the main effects are indicated in the text only.

## Results

### Hypoglycaemia

**Figure 1:**
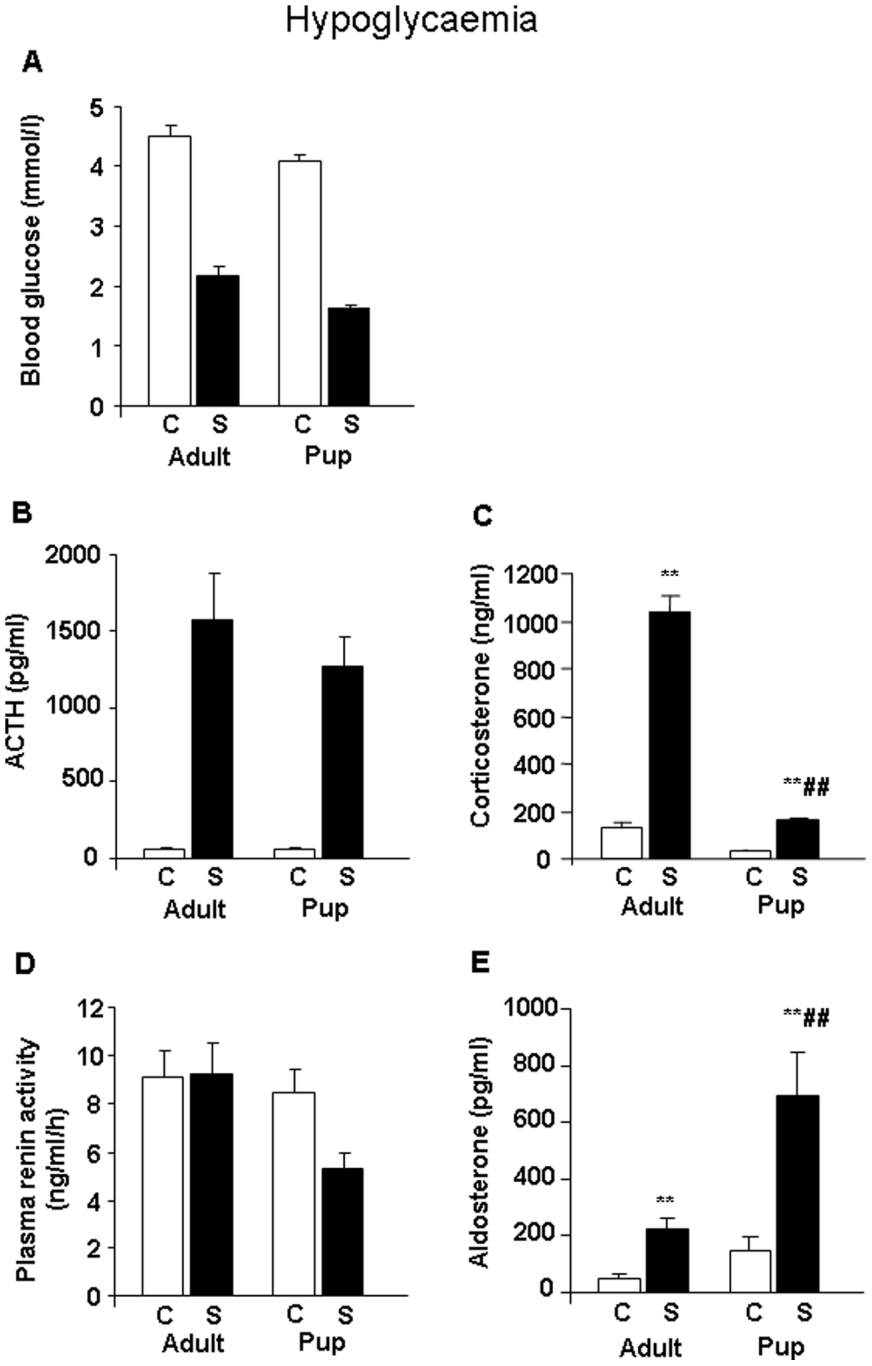
Changes in plasma hormone and glucose concentrations during stress of hypoglycaemia in male Wistar rats (n = 12–16) at 90 minutes after the insulin injection. A. Blood glucose (mmol/l) – the effect of treatment and age were statistically significant (p<0.01). B. Plasma ACTH (pg/ml) – the effect of treatment was statistically significant (p<0.01). C. Plasma corticosterone (ng/ml) – the effects of age (p<0.01), treatment (p<0.01) and age x treatment (p<0.01) interaction were statistically significant. D. Plasma renin activity (ng/ml/h) – the effect of age (p<0.05) was statistically significant. E. Plasma aldosterone (pg/ml) – the effects of age (p<0.01), treatment (p<0.01) and age × treatment (p<0.05) interaction were statistically significant. Abbreviations: C: control, unstressed; S: stressed; **p<0.01 vs. appropriate control group; ##p<0.01 vs. appropriate adult group.

Blood glucose levels decreased significantly at 60 minutes (treatment: F_(1,29)_ = 223.00, p<0.01) (Table 1), and 90 minutes (treatment: F_(1,49)_ = 524.0, p<0.01) (Fig. 1A) following insulin injection. Despite the overall lower blood glucose in pups (age: F_(1,50)_ = 12.1, p<0.01) there was no significant difference in the degree of hypoglycaemia observed in postnatal and in adult rats (no significant interaction between age x treatment). Concentrations of ACTH increased in the stressed groups (at 60 min: treatment F_(1,43)_ = 4.53, p<0.01) (Table 1); (at 90 min: treatment F_(1,49)_ = 58.04, p<0.01) (Fig. 1B) in a similar manner in postnatal and in adult animals at both studied timepoints. Plasma corticosterone levels increased during hypoglycaemic stress, but the elevation was smaller in pups compared to adults at both 60 (age F_(1,43)_ = 311.96, p<0.01; treatment F_(1,43)_ = 305.84, p<0.01; age x treatment F_(1,43)_ = 254.96, p<0.01) (Table 1) and 90 minutes (age F_(1,49)_ = 202.63, p<0.01; treatment F_(1,49)_ = 233.80, p<0.01 age × treatment F_(1,49)_ = 130.70, p<0.01) (Fig. 1C) following insulin injection. Hypoglycaemia had no effect on plasma renin activity, which was lower in pups compared to adults (age F_(1,49)_ = 5.59, p<0.05) (Fig. 1D). On the other hand, hypoglycaemia resulted in a significant rise in aldosterone concentrations in both age groups not only at 60 min (treatment F_(1,43)_ = 18.96, p<0.01) (Table 1), but also 90 min after insulin injection (treatment F_(1,49)_ = 16.95, p<0.01) (Fig. 1B). At 90 min, plasma aldosterone response to insulin injection in 10-day-old rats was significantly higher compared to that in adult animals (age F_(1,49)_ = 10.46, p<0.01; age × treatment F_(1,49)_ = 4.51, p<0.05) (Fig. 1B).

**Table 1:** Hypoglycaemic stress: sampling 60 min after treatment.

	Adult (3 months old)		Pup (10-day-old)	
	Control	Hypoglycaemia	Control	Hypoglycaemia
Blood glucose (mmol/l)	6.2±0.3	3.1±0.2	4.3±0.1	2.1±0.1
ACTH (pg/ml)	92.5±34.8	2942.1±485.1	120.2±21.7	2252.9 ±440.8
Corticosterone (ng/ml)	56.3±7.9	653.2±45.0**	26.0±1.8	53.2±3.9##
Aldosterone (pg/ml)	24.2±4.8	161.7±15.5	46.1±11.1	227.1±63.1

Blood samples were collected 60 minutes after insulin injection. The blood glucose level decreased (treatment p<0.01) and the ACTH level increased in the treated groups (treatment p<0.01). The corticosterone level increased after insulin-induced hypoglycaemia, but this elevation was significantly lower compared to adults (age p<0.01; treatment p<0.01 age × treatment p<0.01; treated adults vs. treated juveniles p<0.01). The aldosterone level was elevated after insulin injection in both age groups (treatment p<0.01). *p<0.05, **p<0.01 vs. Control; ##p<0.01 vs. Adult.

### Immune challenge

**Figure 2:**
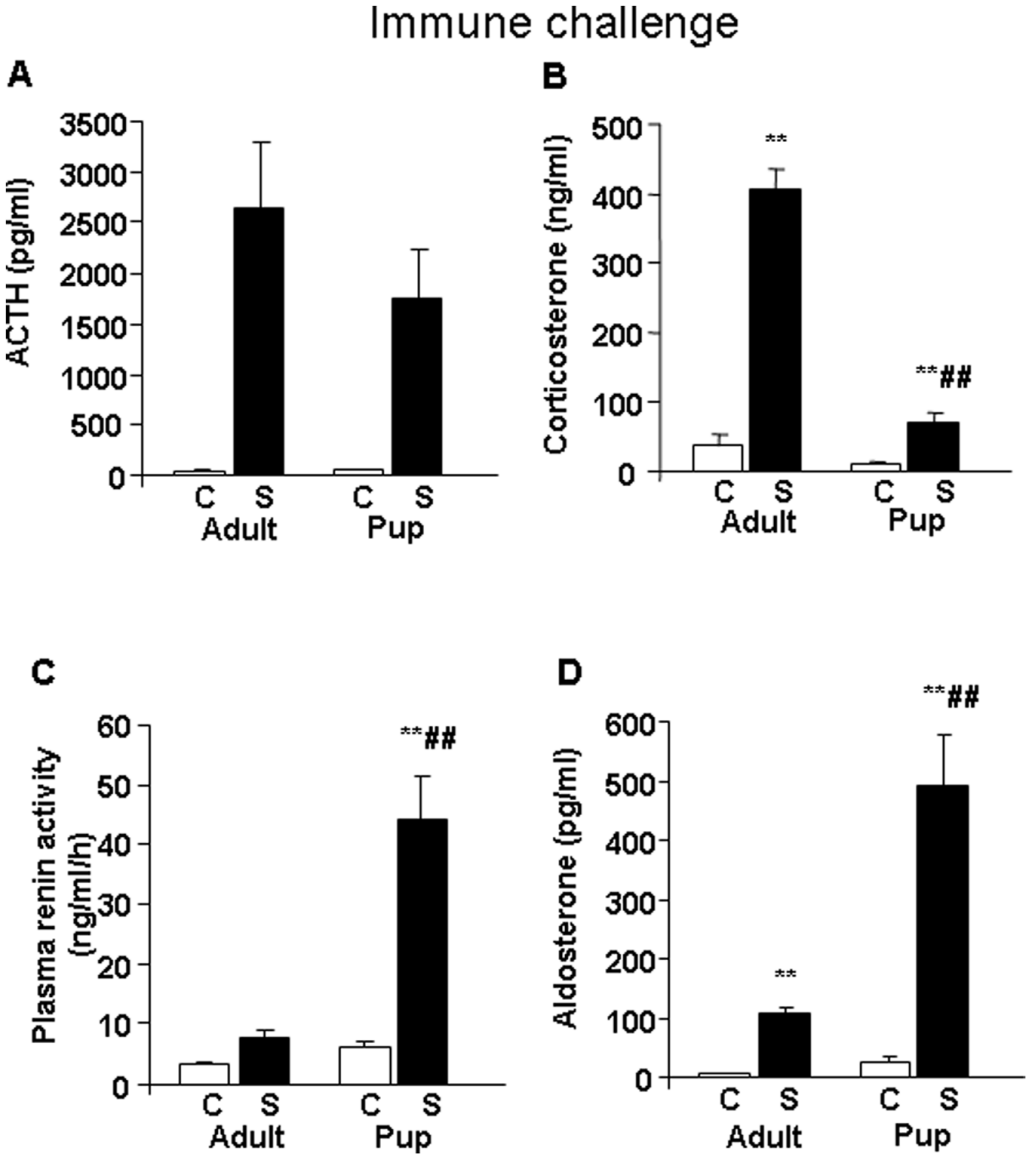
Changes in plasma hormone concentrations during stress of immune challenge at 120 minutes after lipopolysaccharid (LPS) treatment in male Wistar rats (n = 8–10). A. Plasma ACTH (pg/ml) – the effect of treatment was statistically significant (p<0.01). B. Plasma corticosterone (ng/ml) – the effects of age, treatment and age x treatment interaction were statistically significant (p<0.01). C. Plasma renin activity (ng/ml/h) – the effects of age, treatment and age × treatment interaction were significant (p<0.01). D. Plasma aldosterone (pg/ml) – the effects of age, treatment and age × treatment interaction were significant (p<0.01). Abbreviations: C: control, unstressed; S: stressed; **p<0.01 vs. appropriate control group; ##p<0.01 vs. appropriate adult group.

Hormonal changes induced by LPS treatment were very similar to those observed during hypoglycaemia. Plasma ACTH levels increased in response to the immune stressor in both age groups (treatment F_(1,33)_ = 31.90, p<0.01) (Fig. 2A). Concentrations of plasma corticosterone during stress were significantly elevated in both age groups, but the elevation was much lower in the pups (age F_(1,33)_ = 784.84, p<0.01; treatment F_(1,33)_ = 1120.68, p<0.01; age × treatment F_(1,33)_ = 592.04, p<0.01) (Fig. 2B). Plasma renin activity increased in response to immune challenge (treatment F_(1,33)_ = 41.74, p<0.01), exhibiting the highest values in LPS treated pups, which were significantly different from those in LPS injected adults (age F_(1,33)_ = 36.10, p<0.01; age × treatment F_(1,33)_ = 26.12, p<0.01) (Fig. 2C). Changes in plasma aldosterone levels were similar to those in plasma renin activity, however, in contrast to the changes in corticosterone release, the elevation in plasma aldosterone was significantly higher in pups compared to that in adults (age F_(1,33)_ = 30.96, p<0.01; treatment F_(1,33)_ = 60.64, p<0.01; age × treatment F_(1,33)_ = 24.55, p<0.01) (Fig. 2D).

### Receptor and enzyme mRNA levels

**Figure 3:**
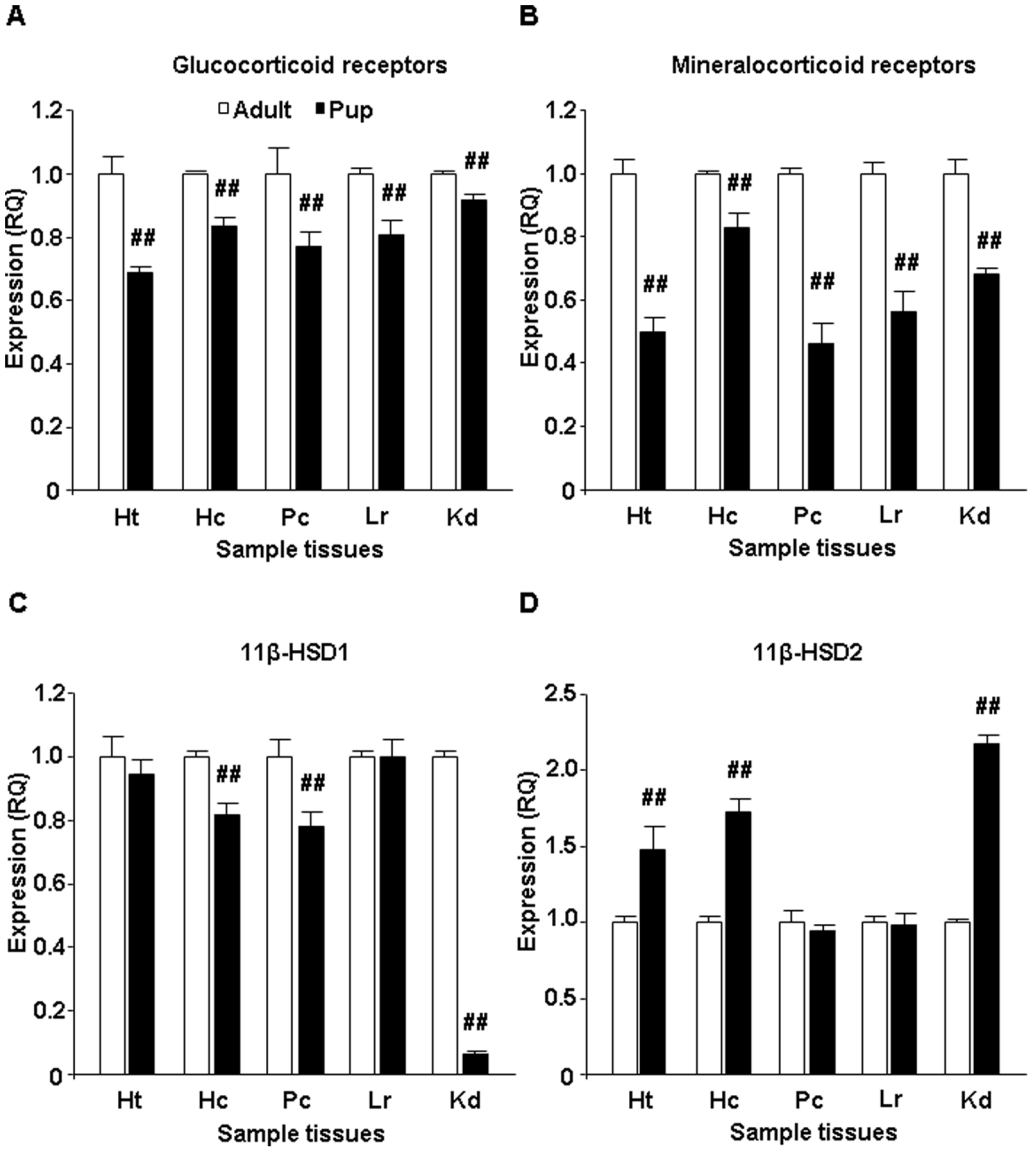
Glucocorticoid receptor (GR), mineralocorticoid receptor (MR), 11-beta-hydroxysteroid dehydrogenase 1 and 2 (11β-HSD1 and 11β-HSD2) mRNA levels in the hypothalamus, hippocampus, prefrontal cortex, liver and kidney under non-stress conditions (n = 4). A. GR mRNA – the effect of age was statistically significant on all studied area (p<0.01). B. MR mRNA – the effect of age was significant on all studied area (p<0.01). C. 11β-HSD1 mRNA – the effect of age was statistically significant in the hippocampus, prefrontal cortex and kidney (p<0.01). D. 11β-HSD2 mRNA – the effect of age was significant in the hypothalamus, hippocampus and kidney (p<0.01). Abbreviations: Ht: Hypothalamus; Hc: Hippocampus; Pc: Prefrontal cortex; Lr: Liver; Kd: Kidney; ##p<0.01 vs. appropriate adult group.

The resting levels of GR mRNA were lower in 10-day-old rats compared to those in adults in all tissues studied (hypothalamus: F_(1,4)_ = 155.27, p<0.01; hippocampus: F_(1,4)_ = 329.69, p<0.01; prefrontal cortex: F_(1,4)_ = 39.80, p<0.01; liver: F_(1,4)_ = 95.89, p<0.01; kidney: F_(1,4)_ = 76.21, p<0.01) (Fig. 3A). Similarly, MR mRNA levels in pups were lower in comparison with those observed in adults (hypothalamus: F_(1,4)_ = 385.75, p<0.01; hippocampus: F_(1,4)_ = 74.04, p<0.01; prefrontal cortex: F_(1,4)_ = 412.66, p<0.01; liver: F_(1,4)_ = 215.62, p<0.01; kidney: F_(1,4)_ = 227.99, p<0.01) (Fig. 3B).

Tissue specific differences were revealed in the mRNA levels of 11β-HSD1 and 11β-HSD2. In the brain, 11β-HSD1 mRNA levels were lower in the hippocampus and prefrontal cortex of pups compared to the levels found in adult animals (hippocampus: F_(1,4)_ = 168.39, p<0.01; prefrontal cortex: F_(1,4)_ = 56.65, p<0.01), while there was no age effect in the hypothalamus (Fig. 3C). The highest difference was observed in the kidney with very low concentration of 11β-HSD1 mRNA levels in pups (kidney: F_(1,4)_ = 11092.12, p<0.01) (Fig. 3C). In contrast, concentrations of 11β-HSD2 mRNA were higher in 10-day-old pups in the kidney as well as in brain tissues with the exception of the prefrontal cortex (hypothalamus: F_(1,4)_ =  50.98, p<0.01; hippocampus: F_(1,4)_ =  249.61, p<0.01; kidney: F_(1,4)_ =  1815.96, p<0.01) (Fig. 3D). No age effect in the mRNA levels of both 11β-HSD1 and 11β-HSD2 was found in the liver.

### Receptor protein levels

**Figure 4:**
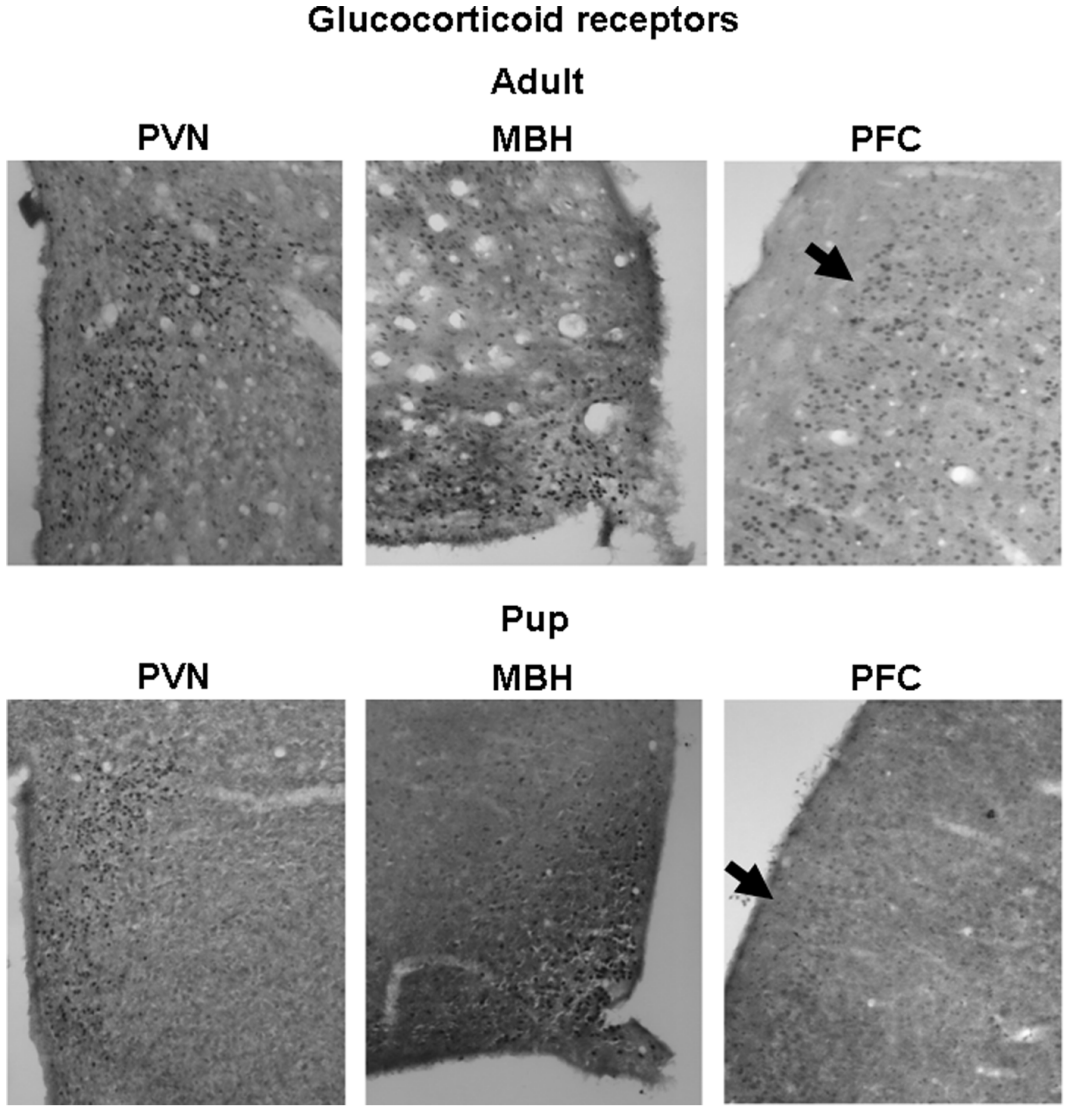
Representative pictures of glucocorticoid receptor immunohistochemistry on adult (approx. 3-month-old) and pup (10-day-old) rat brains. Selected area of the hypothalamus (PVN: nucleus paraventricularis hypothalami, MBH: mediobasal hypothalamus) and prefrontal cortex (PFC) are presented. Arrows represent different distribution of the GR immunoreactivity in adult and pup PFC regions.

**Figure 5:**
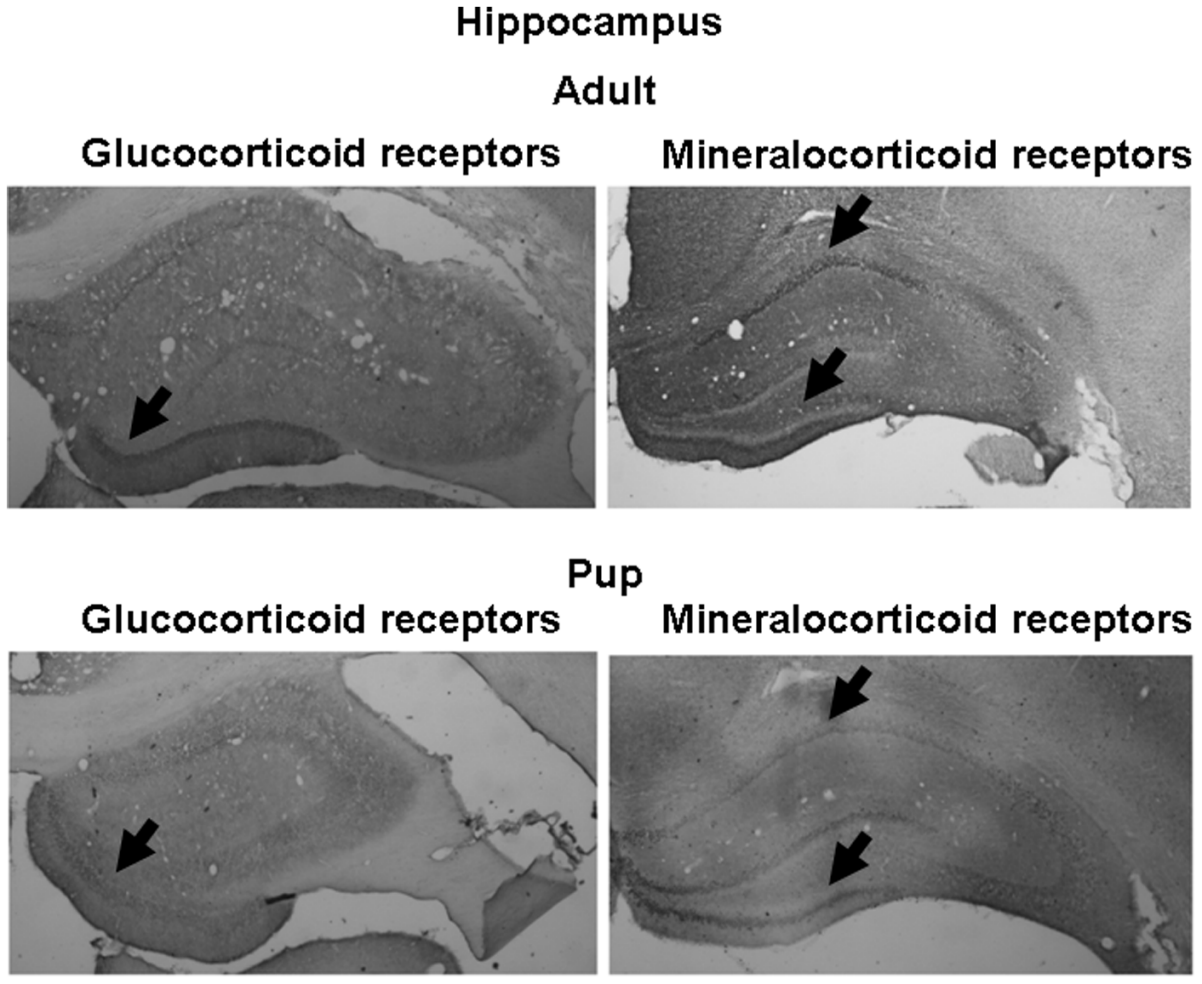
Representative pictures of glucocorticoid and mineralocorticoid receptor immunohistochemistry on adult (approx. 3-month-old) and pup (10-day-old) rat hippocampus. Arrows represent different insensity of immunoreactivity at corresponding areas.

GR immunoreactive (ir) neurons were widely distributed in the brains of both adults and pups. Their distribution in the hypothalamus (nucleus paraventricularis hypothalami and mediobasal hypothalamus) and in prefrontal cortex region is presented in Fig. 4. In accordance with the mRNA data, pups showed smaller number of GR positive cell nuclei than adults in all three brain areas studied. In the prefrontal region, not only the number, but also the distribution was substantially different between the two age groups. In the hippocampus, the highest GRir was found in the dentate gyrus region, being lower in pups compared to adults (Fig. 5).

**Figure 6:**
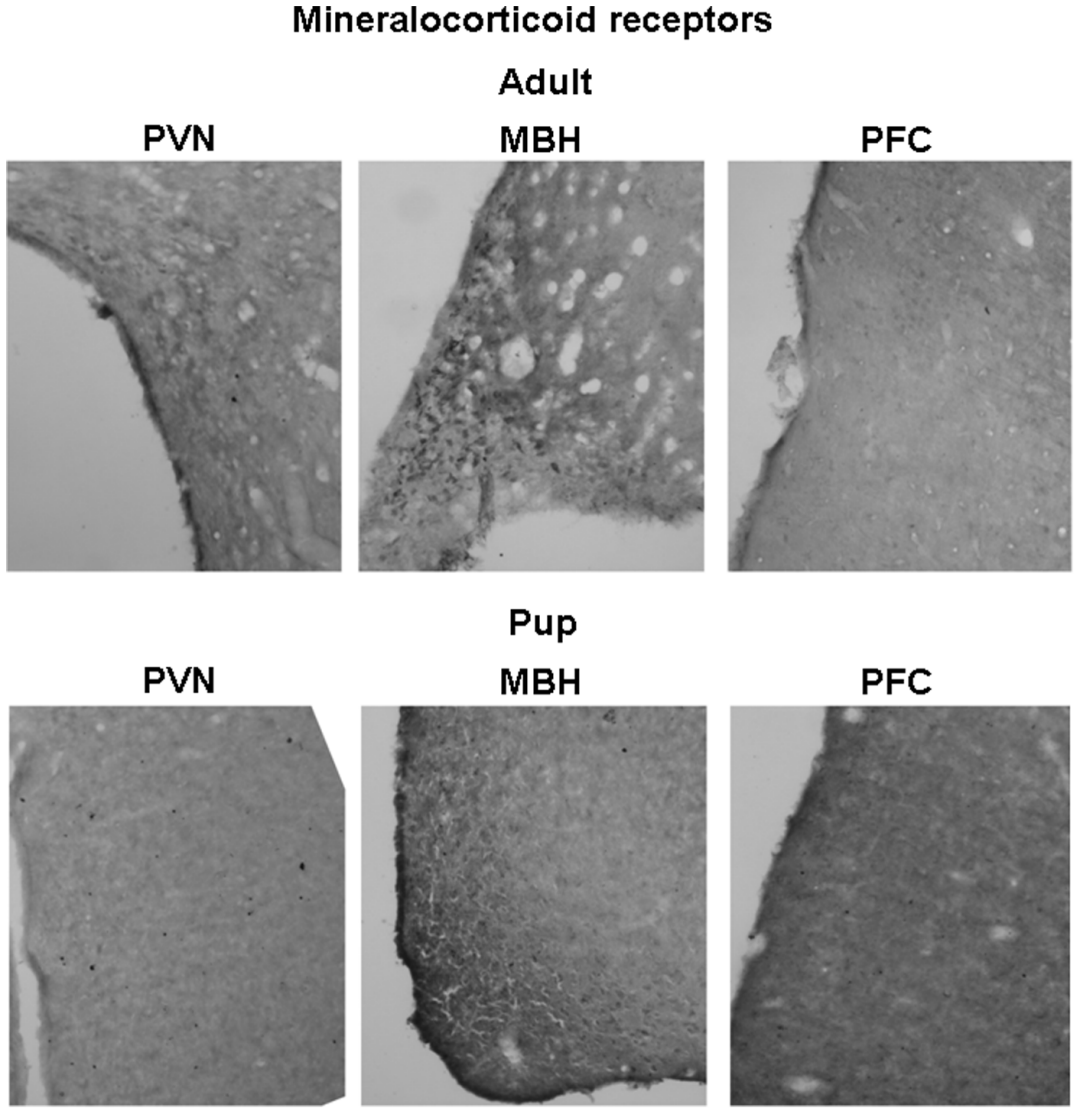
Representative pictures of mineralocorticoid receptor immunohistochemistry on adult (approx. 3 month old) and pup (10-day-old) rat brains. Selected area of the hypothalamus (PVN: nucleus paraventricularis hypothalami, MBH: mediobasal hypothalamus) and prefrontal cortex (PFC) are presented. Few immunoeactive cells are present in the PVN and MBH regions.

The MR proteins were found to be highly expressed in the hippocampal (Fig. 5) and mediobasal hypothalamic (Fig. 6) regions. In the hippocampus, the changes in MRir during the development were site specific. In the CA1 region, the MRir was smaller, while in the region of dentate gyrus it was higher in pups compared to that in adults. In the mediobasal hypothalamic region, the number of MRir positive neurones increased with age (Fig. 6).

### Sensitivity of adrenal cortex to ACTH in vitro

**Figure 7:**
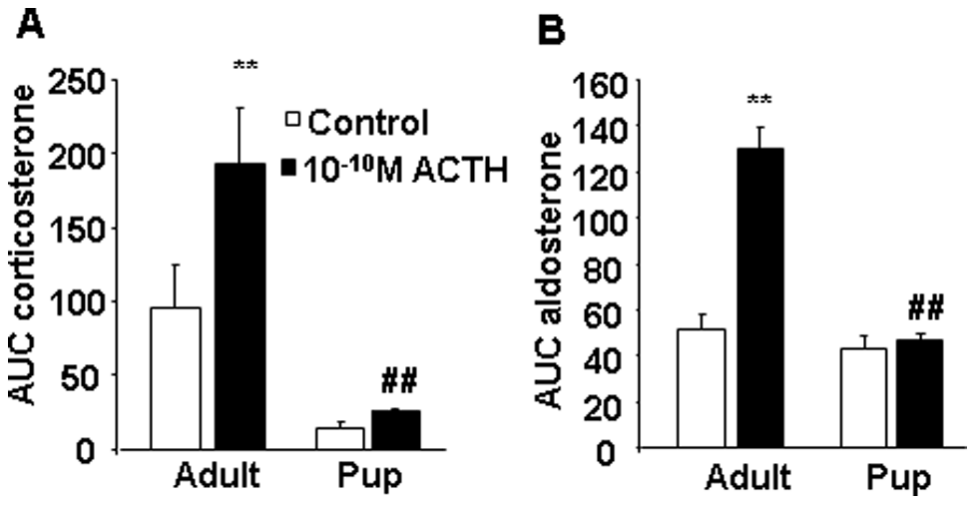
In vitro ACTH sensitivity of adult and pup adrenal gland: secretion of corticosterone (A) and aldosterone (B) (n = 4). After 2 h preincubation we collected 3×15 min fractions with 10^−10^ M ACTH in the second fraction. AUC: area under the curve; the total secreted amount during the whole observation period. **p<0.01 vs. Control; #p<0.05, ##p<0.01 vs. Adults.

The overall corticosterone secretion (AUC) was significantly lower in pups compared to adults during the whole observation period (age: F_(1,12)_ =  26,8, p<0.01) (Fig. 7A). Administration ACTH to the incubation medium significantly elevated the secreted amount of corticosterone (treatment: F_(1,12)_ = 5.2, p<0.05). However, the ACTH-induced corticosterone secretion was not different in the two age-groups (no interaction between age × treatment).

Regarding aldosterone secretion, a similar general diminution was visible in pups (age: F_(1,9)_ = 34.8, p<0.01) (Fig. 7B). ACTH administration induced a significant effect (treatment: F_(1,9)_ = 27.4, p<0.01), which was detectable only in adults (age × treatment: F_(1,9)_ = 22.45, p<0.01).

## Discussion

**Figure 8:**
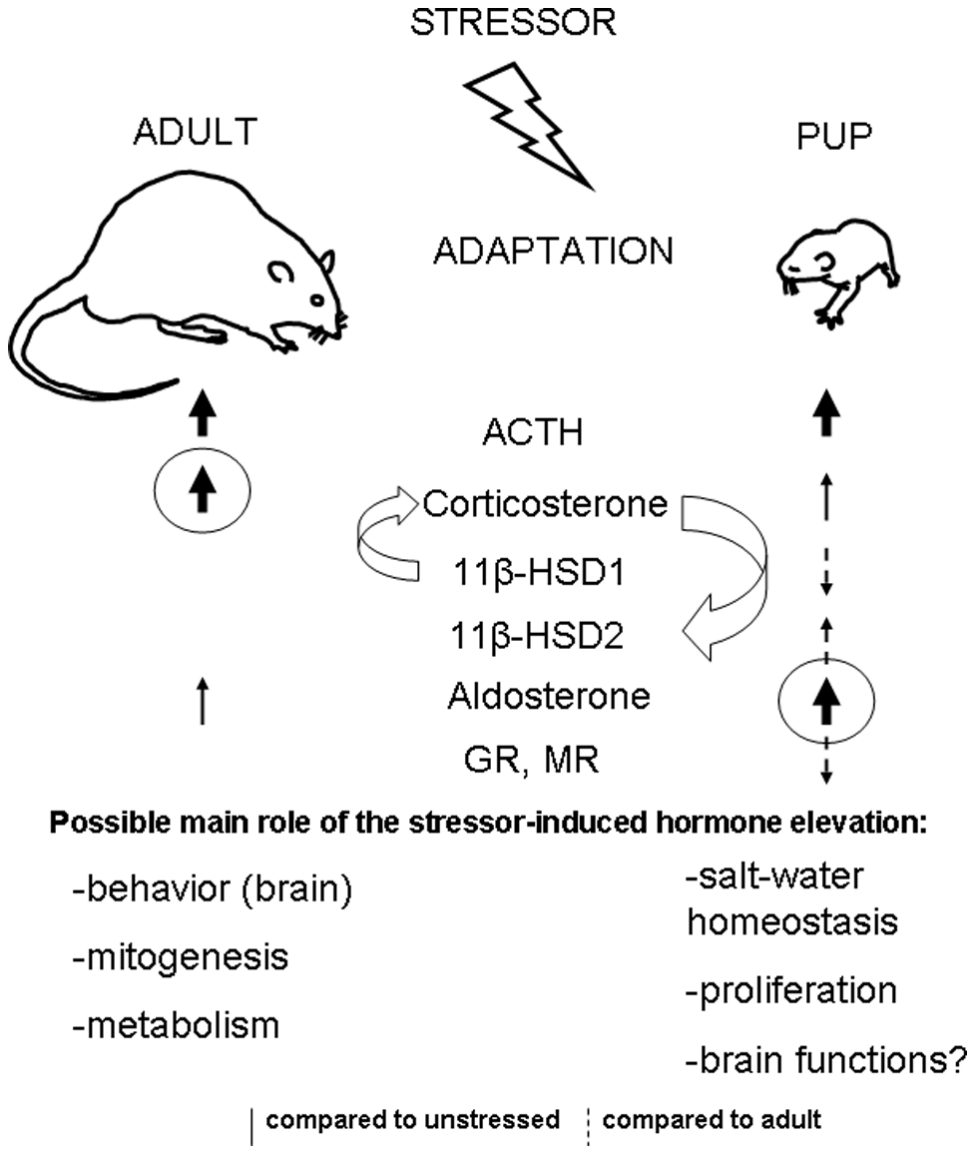
Summary of the gluco- and mineralocorticoid differences during development. Adaptation to stress conditions is fundamental component of survival but the mechanisms seem to be different at different developmental stages (e.g. in adults and in pups). Stressors activate adrenocorticotropin (ACTH) secretion from the pituitary in both adults and pups. However, at the level of the adrenal gland, stimulation of corticosterone secretion is more pronounced in adults compared both to those in pups and to that of aldosterone secretion, while the opposite (namely more pronounced activation of aldosterone secretion) is present in pups. The lower glucocorticoid synthesizing 11β-hydroxysteroid dehydrogenase (11β-HSD1) and gluco- (GR) and mineralocorticoid receptor (MR) mRNA levels in pups seem to be protective mechanisms, preventing the organs (especially the brain) of the pups from excessive glucocorticoid effects. On the other hand, enhanced glucocorticoid degrading 11β-HSD2 expression may enable increased mineralocorticoid effects during the perinatal period.

Present data provide the first evidence of increased aldosterone responses to stress stimuli during the postnatal period suggesting a shift in the balance between stress-induced glucocorticoid and mineralocorticoid hormone release during the development (Fig. 8). In 10-day-old rat pups, which exhibited the well known reduction in stress-induced corticosterone release during SHRP, stress-induced elevation in aldosterone concentrations were significantly higher compared to those in adulthood. Based on the data obtained it seems unlikely that this phenomenon is stressor-specific or valid for only a single time point. Greater importance of mineralocorticoid compared to glucocorticoid actions in postnatal period are further supported by the present observation of increased renal and brain gene expression of 11β-HSD2, an enzyme enabling preferential effects of aldosterone on MR.

Dampened stress-induced corticosterone concentrations in 10-day-old pups and facilitated aldosterone release were revealed using two different stress models (hypoglycaemia, LPS), and different timing. The relatively low corticosterone release in response to stress stimuli is well described and it represents the essential characteristics of stress-hyporesponsiveness during the postnatal period [15], [22]–[24]. The present results are strongly supported by the findings obtained by Raff et al. [30] in 7-day-old pups. Mentioned authors found approx. 10 fold elevation of plasma aldosterone to prolonged (7 days) hypoxia without changes in plasma renin activity, while the accompanied rise in corticosterone levels was only approx. 2 fold [30], though this pattern was not fully comfirmed in a later study of the same group [31]. No comparisons beween the responses in pups and adults were made in these studies, however, the work of Raff et al [30] provided a clear demonstration of increased aldosteronogenesis during early postnatal period. In addition, in human infants aged 3–4 months, ACTH-stimulated aldosterone secretion (3 fold) was higher than that of cortisol secretion (1.5 fold) [32]. Interestingly, it is known for a long time that basal, non-stress aldosterone concentrations in plasma are declining during infancy and childhood in humans [29]–[31].

The secretion of ACTH, which is an important secretagogue of aldosterone during stress, was similar in pups and adults in both stress models. During immune challenge, the other physiological regulator, angiotensin II [4], could have been the triggering factor of enhanced aldosterone release in pups as indicated by high increase in plasma renin activity in stressed pups but not adults. The magnitude of LPS stimulated values of plasma renin activity in pups is very high encouraging further research using other stimuli at this age of the development. However, we failed to observe an increase in plasma renin activity during hypoglycaemic stress, which is in accordance with previous findings showing that hypoglycaemia is not associated with an elevation of plasma renin activity or noradrenaline release in both humans and rats [32], [33]. In this experiment, basal values of plasma renin activity were rather high. This appears to be a consequence of necessary fasting included in the procedures. Fasting per se may induce an increase in plasma renin activity [34], which could contribute to the non-responsiveness to subsequent hypoglycaemia. However, during hypoglycaemia, ACTH may play a more important role than angiotensin II in triggering aldosterone release. Nevertheless, we cannot conclude that in pups the regulatory influence of ACTH on aldosterone release is superior to angiotensin II, as ACTH concentrations were not higher and the in vitro sensitivity of adrenals to ACTH was not increased in pups compared to adult animals. Moreover, the in vitro data do not support the hypothesis that ACTH predominantly stimulates aldosterone and not corticosterone secretion during the perinatal period. It cannot be excluded, however, that evaluation of responses to several doses of ACTH would reveal some differences. The involvement of other peptides influencing adrenal aldosterone release, such as leptin [35] or Homer 1 [36], [37] may also be considered, but the exact mechanisms leading to enhanced stress-induced aldosterone release during the perinatal period remain to be elucidated.

Potential functional significance of enhanced aldosterone activity during postnatal period is supported by concomitant higher 11β-HSD2 and/or lower 11β-HSD1 mRNA levels in the kidney and several brain areas of pups compared to adults. The present study clearly shows increased mRNA expression of 11β-HSD2 and decreased expression of 11β-HSD1, GR and MR in the brain and kidney of 10-day-old pups compared to that in adult rats. In consistence with these findings, immunohistochemical analysis revealed similar differences in GR and MR protein levels. Developmental changes in gene expression and activity of 11β-HSD2 in some brain areas and peripheral tissues were reported during both fetal and postnatal periods [38]–[40]. In accordance with the present data, GR mRNA in human [41], mouse [42] and rat [43], [44] brain as well as GRir in zebra finch brain [45] were repeatedly reported to increase during postnatal development. In the hippocampus the MR mRNA levels in mouse [42] as well as MRir levels in rat (Fig. 5) seem to show site-specific developmental changes. Positive developmental impact of low glucocorticoid levels as well as their receptors in early development has been recognized for a long time [18], [19], [46]. Similarly, 11β-HSD2 is thought to protect immature mitotically-active brain cells from exposure to potentially deleterious high levels of glucocorticoids [47], [48]. Apparently, higher basal and stress-induced mineralocorticoid secretion observed in the present study is needed to meet the specific demands of the developing organism. Though aldosterone may influence brain functions in adult animals [12], its action during early development remains to be elucidated.

It is suggested that the main physiological role of enhanced aldosterone secretion in pups is related to the maintenance of water-electrolyte balance [49]. The control of blood volume is considerably more tenuous in the newborn than in adults [50]. Despite low MR mRNA concentrations in pups, locally increased expression of 11β-HSD2 may provide sufficient aldosterone action. Moreover, aldosterone may provide protection from cell volume swelling via non-genomic mechanisms as observed in the pup kidney as well as during lung oedema [51]–[53]. Another possibility is that in pups aldosterone may, at least to some extent, act via GR as suggested [54]. Nevertheless, our attempts to find glucocorticoid-like negative feedback effect of aldosterone in pups have failed (data not shown).

Furthermore, MRs are present also in the heart, blood vessels, adipose tissue and macrophages [55]. Studies using the aldosterone antagonist spironolactone have revealed that aldosterone might modulate cell proliferation and apoptosis in the neonatal rat heart, so influence cardiac growth and development [56]. It may be suggested that throughout the perinatal period aldosterone overtakes the regulatory role of glucocorticoids in certain cellular processes and molecular mechanisms, particularly those related to stress.

Taken together, a primarily mineralocorticoid regulation under stress conditions at the time of postnatal SHRP may be evolutionarily warranted. Though additional information is needed, the results of the present study clearly show a higher stress-induced release of aldosterone in pups compared to adults and strongly suggest greater importance of mineralocorticoid compared to glucocorticoid actions during the postnatal period.
